# An efficient approach to cathode operational parameters optimization for microbial fuel cell using response surface methodology

**DOI:** 10.1186/2052-336X-12-33

**Published:** 2014-01-14

**Authors:** Mohammadreza Hosseinpour, Manouchehr Vossoughi, Iran Alemzadeh

**Affiliations:** 1 Chemical and Petroleum Engineering Department, Sharif University of Technology, Tehran, Iran; 2 Institute of Biotechnology and Environmental Studies (IBE), Sharif University of Technology, Tehran, Iran

**Keywords:** Microbial fuel cell (MFC), Cathode compartment, Optimization of operational parameters, Response surface methodology (RSM)

## Abstract

**Background:**

In the recent study, optimum operational conditions of cathode compartment of microbial fuel cell were determined by using Response Surface Methodology (RSM) with a central composite design to maximize power density and COD removal.

**Methods:**

The interactive effects of parameters such as, pH, buffer concentration and ionic strength on power density and COD removal were evaluated in two-chamber microbial batch-mode fuel cell.

**Results:**

Power density and COD removal for optimal conditions (pH of 6.75, buffer concentration of 0.177 M and ionic strength of cathode chamber of 4.69 mM) improve by 17 and 5%, respectively, in comparison with normal conditions (pH of 7, buffer concentration of 0.1 M and ionic strength of 2.5 mM).

**Conclusions:**

In conclusion, results verify that response surface methodology could successfully determine cathode chamber optimum operational conditions.

## Background

Microbial fuel Cell was introduced as a novel technology for sustainable electrical energy production from organic waste water [1,2]. Furthermore, this technology could be applied as an alternative process for wastewater treatment instead of activated sludge. MFC technology offer many advantages such as lower sludge production, more effective organic load removal as well as lower net energy consumption, in comparison with activated sludge.

In MFCs, microorganisms in anode chamber act similar to metal catalysts in chemical fuel cells and consume soluble organic components. In the absence of ultimate electron acceptor, generated electrons could be transferred directly or by means of nano-wires or mediators from microorganisms to anode electrode. On the other hand, hydrogen ions which are generated in microbial metabolic reaction are transferred to cathode compartment across proton exchange membrane such as Nafion 117 Dupont. Then, electrons, hydrogen ions and oxidant react on the cathode electrode. Continuous electron transfer from anode to cathode electrode is necessary for the completion of the aforementioned reaction in MFCs. When MFCs operate ideally, they can produce electricity as long as the substrate is supplied. The ideal voltage of the cell can be thermodynamically predicted by the Nernst equation (1) [1,3]:

(1) $$E_{\mathrm{thermo}}=E^{0}-\frac{\mathrm{RT}}{\mathrm{nF}}\ln\left(\pi \right)$$

Where *E*^0^ is the standard cell potential (V), *R* is the ideal gas constant (8.314 J/mol.K), *T* is the absolute temperature (K), *n* is the number of electrons transferred in the reaction (dimensionless), *F* is the Faraday’s constant (96,485C/mol), and Π is the chemical activity of the products divided by those of the reactants (dimensionless). In fact, the nature of substrate and its concentration as well as temperature could affect MFC performance.

In practice, the actual voltage of MFC is lower than the value thermodynamically predicted, owing to three distinct factors in anode and cathode compartments. The factors are activation overpotential, ohmic loss and concentration overpotential [1,3]. The actual voltage of MFC could be determined by subtracting all overpotentials from thermodynamically predicted cell voltage as follows [4]:

(2) $$\begin{array}{c} v=E_{\mathrm{thermo}}-[{\left(\eta_{\mathrm{act}}+\eta_{\mathrm{ohmic}}+\eta_{\mathrm{conc}}\right)}_{\mathrm{cathode}}\\ \;+{\left(\eta_{\mathrm{act}}+\eta_{\mathrm{ohmic}}+\eta_{\mathrm{conc}}\right)}_{\mathrm{anode}} \end{array}$$

Where *E*_thermo_ is the thermodynamically predicted voltage, *η*_*act*_ is the activation loss due to reaction kinetics, *η*_*ohmic*_ is the ohmic loss from ionic and electronic resistances, and *η*_*conc*_ is the concentration loss due to mass transport limitations. The equation (2) implied that losses in both anode and cathode chambers would lead to a cell voltage reduction.

As a result, optimization of both anode and cathode compartments could decrease the overpotentials and consequently improve cell voltage as well as electrical power production.

First of all, activation overpotential relates to the rates of reactions on the anode and cathode electrode. The activation energy reduction of anodic or cathodic reactions by means of catalysts could boost cell output. Pt alloys were used extensively and showed enhancement in energy production in MFCs [5]. Due to high cost of Pt, other metal surfaces such as gold, iridium, iron and rhodium were applied in cathode compartment [6]. Among them, transition metal complexes, particularly those based on cobalt (Co) and manganese (Mn) showed promises [7].

Secondly, ohmic overpotentials relate to the resistance of ions flow across the electrolyte and electrons flow through the electrodes and connection materials. To reduce these losses, three major resistances in the cells should be considered. The most important one is caused by ion transfer in the anolyte and catholyte. Mohan et al. investigated the effect of ionic strength on microbial fuel cell performance, he determined that there was an optimum ionic strength that power density reached its maximum value [8]. Huang et al., found that an increase in ionic strength could improve power production due to a decrease in internal resistance [9].

The second ohmic resistance is related to the electrodes as well as connection materials. Application of electrode materials with good electrical conductivity and higher surface area such as copper, carbon and platinum would contribute to lessen the ohmic resistance. Sharma et al., used granular carbon active and carbon clothe as electrode material and found that granular carbon active was 19 times better than carbon clothe [10]. The last ohmic resistance is caused by a membrane which separates anode chamber from cathode cell. Pant et al. utilized new membrane Zirfon to replace high cost Nafion membrane. They also determined Oxygen mass transfer coefficient of Zirfon was comparable with Nafion [11]. For more support, there is a good review of recent advances in separator for Microbial fuel cell [12].

Finally, third overpotential is related to mass transfer resistance in both anode and cathode compartments. To increase mass transfer coefficient, Scott et al. [13] used tubular microbial fuel cell. Liang et al. utilized three different configurations to have better idea of internal resistances distribution [14]. Manohar et al. found that a rise in inlet flow of anode and cathode solution could decrease mass transfer resistance and improve power production [15].

Determination of optimal conditions in MFCs requires extensive exploration of the operating parameters which affect the power production of MFCs. In this study, the optimal values of pH, buffer concentration and ionic strength of cathode electrolyte were determined by Response Surface Methodology (RSM). Response Surface Methodology is a technique to design experiments, evaluate the effects of operating conditions and achieve the best conditions for desirable responses with a limited number of planned experiments [16-19]. To the knowledge of the authors, this is the first study deals with optimization of operational parameters and their interactions in the cathode compartment of microbial fuel cell.

## Methods

### Microbial fuel cell

**Figure 1:**
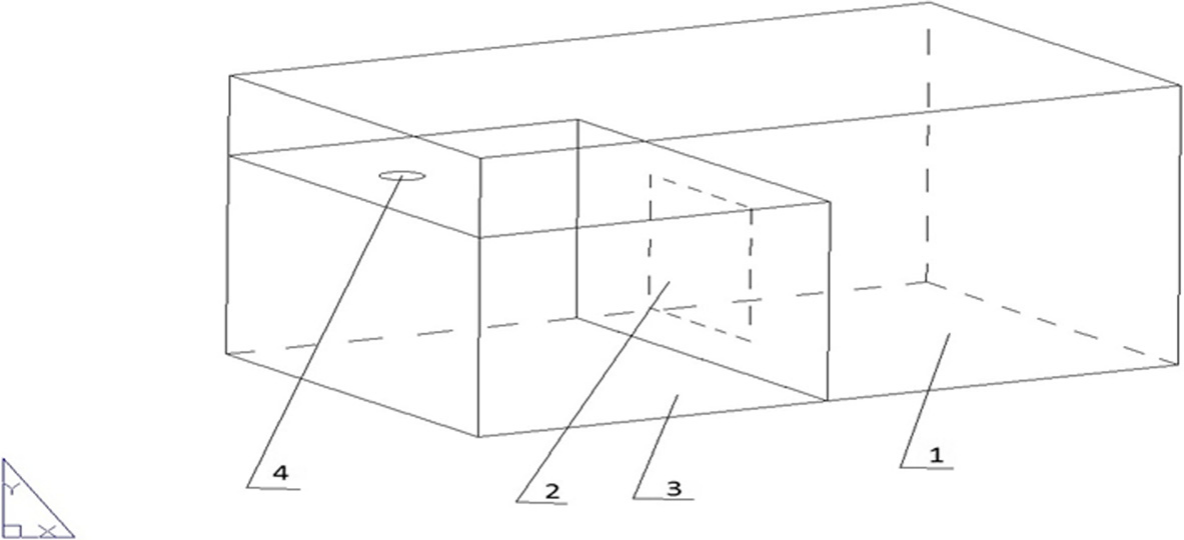
Schematic drawing of designed MFC.1-cathode chamber; 2-proton exchange membrane; 3-anode chamber; 4-sampling port.

A dual-chamber MFC was constructed with following specifications (Figure 1):

– Anode chamber volume: 90 ml (Totally placed in cathode chamber)

– Cathode chamber volume: rectangular prism (dimension = 8 × 6 × 5 cm^3^, Total volume = 240 ml)

– Anode electrode specification: Graphite brush (length = 2.5 cm, diameter = 2.5 cm, total surface area = 0.89 m^2^) was connected to an external resistor of 3300 Ohm with copper wire

– Cathode electrode specification: graphite sheet (area = 2 × 3 cm^2^ and thickness = 0.5 cm) was connected to an external resistor of 3300 Ohm with copper wire

– Membrane: Nafion_ 117 Dupont (surface area = 4.00 cm^2^, thickness = 0.178 mm)

### Wastewater

The growth synthetic media (per liter) was prepared for all experiments consisted of following components: 1000 mg glucose, 0.114 mg of urea,0.046 mg of K_2_HPO_4_, 0.4 mg of FeCl_3_, 3 mg of MgSO_4_, 0.11 mg of CuSO_4_.5H_2_O, 0.7 mg of NaCl, 0.015 mg of ZnCl_2_, 4 mg of Na_2_S_2_O_5_, 0.254 mg of MnSO_4_ and 2.06 mg of FeSO_4_.7H_2_O. The medium was adjusted to pH of 7 and flushed with N_2_ in order to remove oxygen. In all experiments chemical oxygen demand of waste water was fixed on 1160 ppm.

### MFC operation

Anaerobic sludge was supplied by dairy manufacturer (Pegah Co. Tehran, Iran). The sludge was filtered with 20 micrometre filter to remove insoluble particles and then diluted 10 times by the same synthetic medium used for MFC operation. After dilution, medium was heated in order to deactivate methanogensys and kept at 35°C incubator for one week. Later, it was subcultured 3 times in the same medium before inuculation. The prepared sludge was used for MFC inoculation. Catholyte was phosphate buffer which its pH and concentration were determined based on experimental design. NaCl was used to change the ionic strength of cathode electrolyte. In the cathode compartments, oxygen was applied as an oxidant. The total working volume of the anode and cathode were 80 ml and 100 ml, respectively.

### Experimental design

Cathode compartment design is the most important limiting part of power producing MFCs because of poor kinetics of oxygen reduction reaction due to neutral pH conditions and high internal resistance due to low ion concentration in buffer. To overcome these obstacles, optimization of catholyte pH for higher reaction rate and buffer concentration and ionic strength for reduction in internal resistance are the main focus of this article.

In this study, twenty experiments were designed (= 2^k^ + 2 k + 6) respected to three independent variables (k = 3). Then, eight of the experiments (2^3^) are factorial design, six of them (2*3) are axial points (coded ± α) and finally six of experiments are replications of the central values (zero level) to determine pure errors. The value of α equals to 1.682 and is calculated by equation (3):

(3) $$\alpha =2^{\frac{n}{4}}$$

Where n is the number of independent variables in the design.

Table 1 demonstrates the range and level of all the variables. Table 2 shows the experimental design of the independent variables for cathode optimization.

**Table 1:** Levels of independent variables used for process optimization

**Symbol**	**Variables**	**Unit**	**Levels**				
			**-1.68**	**-1**	**0**	**1**	**+1.68**
**A**	pH of catholyte	-	5.32	6	7	8	8.68
**B**	Buffer concentration	M	0.065	0.100	0.150	0.200	0.234
**C**	Ionic strength	mM	0.8	2.5	5.0	7.5	9.2

**Table 2:** Central-composite experimental design of independent variables for cathode optimization

**Run**	**Point type**	**Codded factors**			**Actual factors**			**Responses**	
		**A**	**B**	**C**	**A**	**B**	**C**	**R**_**1**_	**R**_**2**_
1	Center	0	0	0	7	0.150	5.0	33	93.4
2	Axial	1.7	0	0	8.68	0.150	5.0	20.1	77.1
3	Fact	-1	1	-1	6	0.200	2.5	31.1	87.2
4	Axial	-1.7	0	0	5.32	0.150	5.0	25.1	83.1
5	Fact	1	-1	-1	8	0.100	2.5	23.5	83.9
6	Center	0	-1	0	7	0.150	5.0	28.7	94
7	Axial	0	0	1.7	7	0.150	9.2	28.7	88.7
8	Fact	-1	-1	0	6	0.100	2.5	27	86.1
9	Fact	1	1	1	8	0.200	7.5	24.9	82.4
10	Center	0	0	0	7	0.150	5.0	32.2	91
11	Center	0	0	0	7	0.150	5.0	31	93.7
12	Axial	0	1.7	0	7	0.234	5.0	32.5	90.1
13	Fact	1	-1	1	8	0.100	7.5	23.7	81.5
14	Axial	0	0	-1.7	7	0.150	0.8	30	91.4
15	Fact	-1	1	1	6	0.200	7.5	31.7	86.9
16	Center	0	0	0	7	0.150	5.0	31.9	93.4
17	Center	0	0	0	7	0.150	5.0	32.4	89.9
18	Fact	-1	-1	1	6	0.100	7.5	29.6	86.5
19	Fact	1	1	-1	8	0.200	2.5	25.7	84.1
20	Axial	0	-1.7	0	7	0.065	5.0	27.4	89.7

(A: pH of catholyte; B: buffer concentration (M); C: ionic strength (mM); R_1_: Power Density (mW/m2); R_2_: COD Removal (%)).

**Figure 2:**
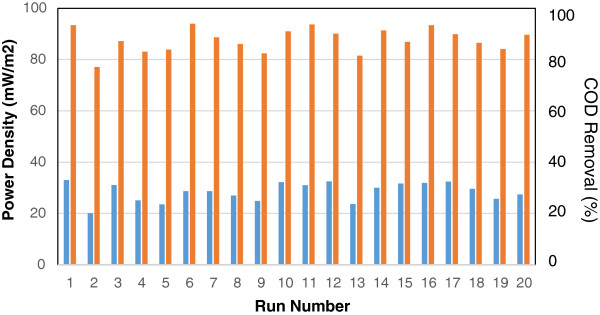
The experimental value of power density (mW/m2) in stationary phase of process and experimental value of COD removal (%) versus run number.

In recent study, power density (mW/m^2^) and chemical oxygen demand (COD) removal (%) were considered as responses of microbial fuel cell under different conditions. Power density and COD removal were analysed by the response surface methodology. Finally, the value of power density in stationary phase of process and final COD removal of MFC for each of twenty designed experiments are depicted in Figure 2.

## Results

### Analysis of variance (ANOVA) and model fitting

ANOVA results of the quadratic model for power density and COD removal present in Tables 3 and 4, respectively. F-value, probability > F, Lack of Fit, and R^2^ are parameters that measure how the quadratic model fit the experimental data. “Adeq Precision” measures the signal to noise ratio and for achievement of desirable result this must be greater than four. Hence, in present quadratic models for power density and COD removal, the ratios of 13.686 and 15.594 indicated adequate signal to noise ratio, respectively.

**Table 3:** Analysis of variance for the quadratic model for power density

**Source**	**Sum of squares**	**Degree of freedom**	**Mean square**	**F-value**	**P-value**	
**Model**	239.07	9	26.56	14.10	0.0001	Significant
**A-Initial pH of catholyte**	65.94	1	65.94	34.99	0.0001	
**B-Catholyte concentration**	24.19	1	24.19	12.84	0.0050	
**C-Ionic strength**	0.013	1	0.013	6.650E-003	0.9366	
**AB**	0.98	1	0.98	0.52	0.4873	
**AC**	1.80	1	1.80	0.96	0.3508	
**BC**	1.12	1	1.12	0.60	0.4576	
**A^2**	141.72	1	141.72	75.21	< 0.0001	
**B^2**	4.16	1	4.16	2.21	0.1682	
**C^2**	8.09	1	8.09	4.29	0.0650	
**Residual**	18.84	10	1.88			
**Lack of fit**	7.05	5	1.41	0.60	0.7070	Not significant
**Pure error**	11.79	5	2.36			
**Total**	257.92	19				
R^2^ = 0.9269		Adj R^2^ = 0. 8612		Adeq precision = 13.686		

**Table 4:** Analysis of variance for the quadratic model for COD removal

**Source**	**Sum of squares**	**Degree of freedom**	**Mean square**	**F-value**	**P-Value**	
**Model**	390.99	9	43.44	19.57	< 0.0001	Significant
**A-Initial pH of catholyte**	45.37	1	45.37	20.44	0.0011	
**B-Catholyte concentration**	0.78	1	0.78	0.35	0.5654	
**C-Ionic strength**	5.34	1	5.34	2.41	0.1519	
**AB**	0.020	1	0.020	9.010E-003	0.9263	
**AC**	2.20	1	2.20	0.99	0.3424	
**BC**	0.000	1	0.000	0.000	1.0000	
**A^2**	321.67	1	321.67	144.92	< 0.0001	
**B^2**	22.87	1	22.87	10.30	0.0093	
**C^2**	20.98	1	20.98	9.45	0.0118	
**Residual**	22.20	10	2.22			
**Lack of fit**	7.90	5	1.58	0.55	0.7343	Not significant
**Pure error**	14.29	5	2.86			
**Total**	413.19	19				
R^2^ = 0.9463		Adj R^2^ = 0.8979		Adeq precision =15.594		

Finally, power density and COD removal were estimated with second-order polynomial equations in terms of coded variables and are given in Eq. (4) and Eq. (5), respectively:

(4) $$\begin{array}{l} \mathrm{Power}\;\mathrm{Density}\;=31.53-2.20\;A+1.33\;B+0.030\;C\;\\ \;-0.35\;A\times B-0.47\;A\times C-0.37\;B\times C\;\\ \;-3.14\;A^{2}-0.54\;B^{2}+0.75\;C^{2}\\ R^{2}=0.926,R_{\mathrm{adj}}^{2}=0.8612,F=14.1\; \end{array}$$

(5) $$\begin{array}{l} \mathrm{COD}\;\mathrm{Removal}\;=92.62-1.82\;A+0.24\;B-0.63\;C\;\\ \;-0.05\;A\times B-0.52\;A\times C-0.000\;B\times C\;\\ \;-4.72\;A^{2}-1.26\;B^{2}-1.21\;C^{2}\\ R^{2}=0.9463,R_{\mathrm{adj}}^{2}=0.8979,F=19.57 \end{array}$$

**Figure 3:**
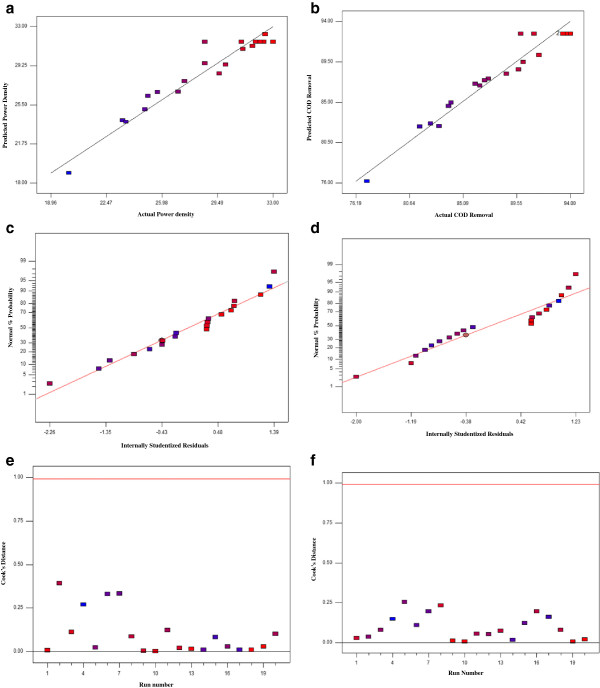
**Analysis of variance.** Actual versus predicted values of power density **(a)** and COD removal **(b)**. Normal plot of residual plots of power density **(c)** and COD removal **(d)**. Cook’s distance plots of power density **(e)** and COD removal **(f)**.

The predicted power density versus experimental data as well as COD removal data are plotted in Figure 3a and b, respectively. The distribution of the majority of experimental data points in the vicinity of the bisection line, indicates the satisfactory correlation between experimental and predicted values. To check the model adequacy studentized residuals which represented the differences between the actual response value and the value that best fitted under the hypothesized model were calculated. The small residual values for both power density and COD removal were specified acceptable accuracy of the model prediction. The normal probability plots of studentized residuals are shown in Figure 3c and d. From these plots it could be concluded that there was no abnormality in this study. According to Figure 3e and f which represent Cook’s distance plots, there was no point that potentially powerful due to the location in the factor for both power density and COD removal.

### Interactive effect of the pH, buffer concentration and ionic strength of cathode compartment on the power density of microbial fuel cell

**Figure 4:**
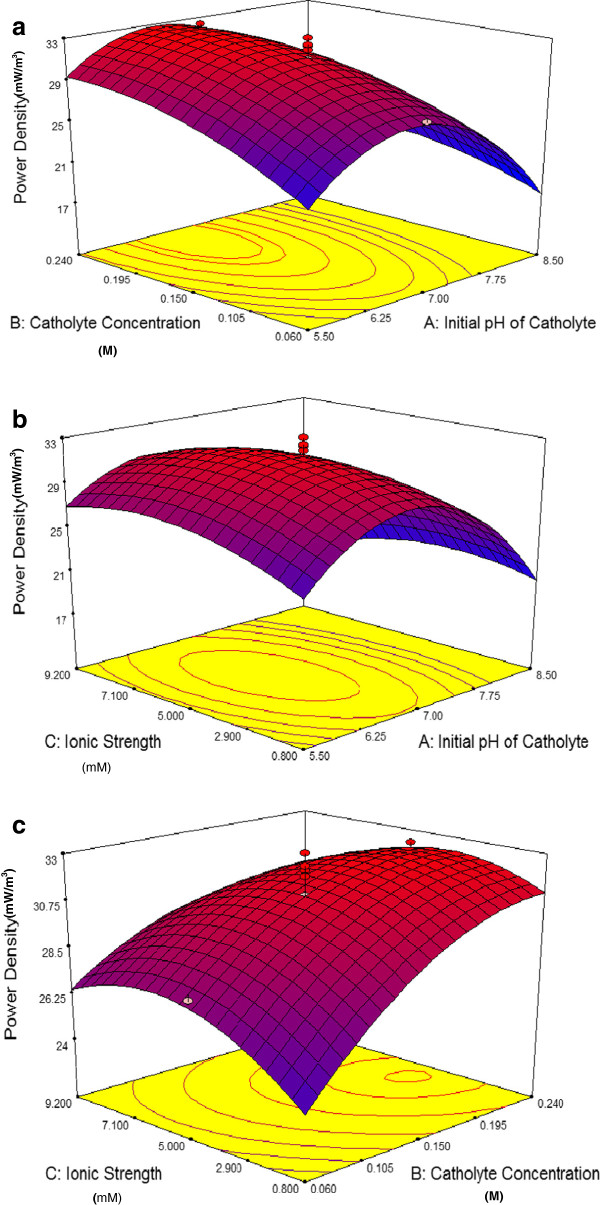
**3D response surface of power density.** Interactive effects of pH and buffer concentration on power density **(a)**; interactive effects of pH and ionic strength on power density **(b)**; interactive effects of buffer concentration and ionic strength on power density **(c)**.

The plots of surface response of power density (Figure 4a-c) were generated while one variable maintained at its zero level, with varying the others within the experimental range.

Figure 4a presents an elliptic characteristic as a result of pH and catholyte concentration interaction. This indicated that both pH and catholyte concentration were influential factors in the design range. As can be seen, the decrease of the initial pH with simultaneous increase in catholyte concentration brings about constant increase of response values to reach the highest level of 33 mW/m^2^. The lower the pH, the higher the hydrogen concentration, the higher the rate of the electrochemical reaction occurs in the cathode chamber. In addition, the rise of buffer concentration share the same effect, contributes to boosting power density production in microbial fuel cell.

When the level of pH decreases to lower than 6.5, this would halt the hydrogen ion transfer from anode chamber to cathode cell as well as acidification of anode compartment. The event would result in death or inactivation of microorganisms leading to reduction of power density as well as COD removal. On the other hand, very high buffer concentrations also may cause salt precipitation on electrodes or membrane which could have an adverse effect on electron and ion transfer. From the response surface plot, the optimal values of pH and catholyte concentration were 6.5 and 0.22 M, respectively when ionic strength was at zero level.

The interaction of initial pH and ionic strength of the cathode solution at zero level of catholyte concentration are shown in Figure 4b. Based on aforementioned figure, there was an elliptic characteristic with the long axis of the ellipse running along the ionic strength axes. This indicated that pH was more influential than ionic strength in the design range. For ionic strengths less than 5.34 mM, a rise in ionic strength has a positive effect on power density production due to higher conductivity. Additional increase of ionic strength, however, might lead to precipitation of salt on electrode or membrane as well as reduction of hydrogen ion transfer to cathode electrode and consequently abatement of power density production in MFC. From Figure 4b, it could be concluded that at zero level of catholyte concentration, the optimum value of pH and ionic strength for power density production were 6.6 and 5.34 mM, respectively. Beyond that point, increasing both pH and Ionic strength would reversely affect the power density production.

Figure 4c demonstrates the influence of catholyte concentration and ionic strength on the power density production. These two factors are in favour of power density production as a result of an increase in conductivity. Power density production was increased gradually when catholyte concentration and ionic strength increased to the level of 0.22 M and 4.20 mM, respectively. From that point, the higher the cathode electrolyte concentration and ionic strength, the lower the power density production would occur as a result of salt precipitation on electrode and membrane. It should be mentioned that at low ionic strength and buffer concentration, power density dramatically drops as a result of low conductivity.

### The interactive effect of the pH of catholyte, buffer concentration and ionic strength on COD removal in microbial fuel cell

**Figure 5:**
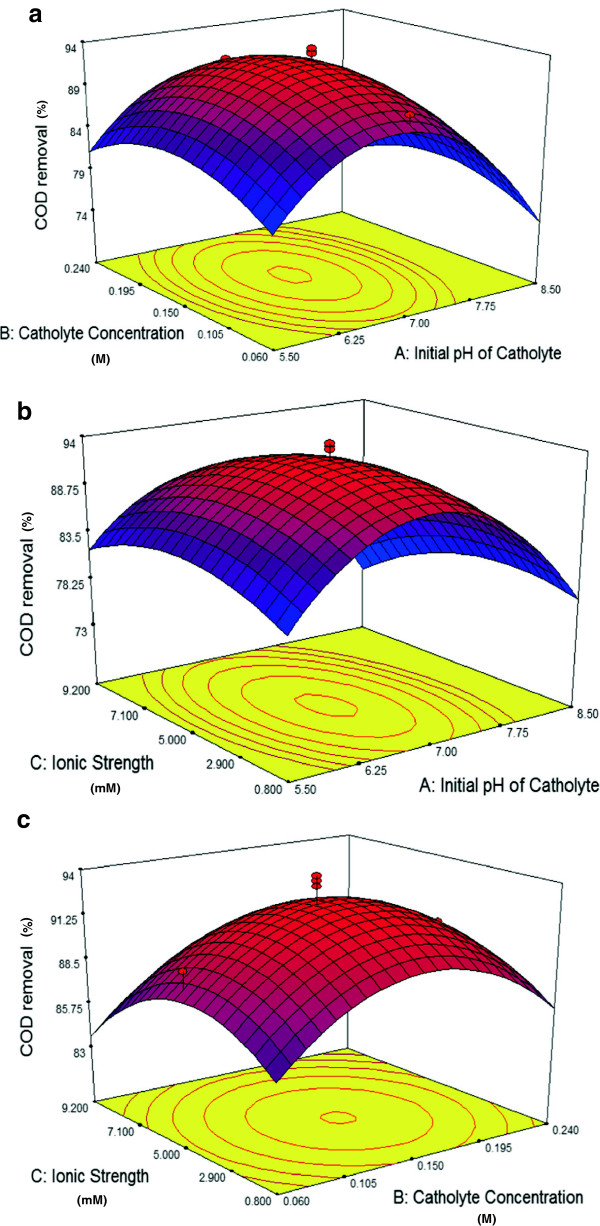
**3D response surface of COD removal.** Interactive effects of Initial pH and catholyte concentration on COD removal **(a)**; interactive effects of initial pH and ionic strength on colour COD removal **(b)**; interactive effects of catholyte concentration and ionic strength on COD removal **(c)**.

Interactive effects of the aforementioned factors on the COD removal were depicted in Figure 5a. Microorganisms in the anode chamber consume glucose as their sole carbon and energy sources and this would lead to generation of electron current and power. Consequently, to increase the rate of the electrochemical reaction as well as power density, higher electron production and substrate consumption are required in anode chamber. It could be expected that trend of COD removal would be similar to power production.

On the other hand, oxygen leakage from cathode to the anode chamber could result in more COD removal without any significant effect on the power production. Substrate, moreover, seeps through a membrane from anode to cathode chamber and water leakage from cathode to anode could affect COD removal in MFC. As a result, COD removal is not as sensitive as power density to variable factors and it varies in a limited range of 83 to 94%.

Figure 5a shows interaction of pH and catholyte concentration on COD removal, with maintaining ionic strength at its zero level of 5 mM. From Figure 5a, it could be concluded that COD removal would improve by lowering initial pH and increasing the buffer concentration. As it was mentioned, the trend of response surface is similar to power density. The maximum COD removal was achieved at the condition with the pH of 6.8 and buffer concentration of about 0.157 M.

Interaction of initial pH and ionic strength while buffer concentration was at its zero level of 0.15 M were shown in Figure 5b. This caused an elliptic characteristic with the long axis of the ellipse running along the ionic strength axes. It could be concluded that the initial pH had more influence on COD removal than ionic strength. Similar to power density, ionic strength could elevate COD removal, yet higher ionic strength (more than 4.51 mM) had adverse effects on COD removal due to salt precipitation on membrane and cathode electrode. Based on response surface plot, low pH led to a decrease in COD removal because of the formation of a hydrogen ion gradient between anode and cathode chamber which prevented H^+^ transfer from anode to cathode compartment. Moreover, pH of higher than 6.8 was not desirable owing to low hydrogen ion concentration in the cathode cell which could complete electrochemical reaction on the cathode electrode.

Figure 5c depicts the interaction of cathode buffer concentration and ionic strength while initial pH was at its zero level of 7.0. Surface response plot indicated that COD removal was more sensitive to catholyte concentration than ionic strength. In fact, the influence of catholyte concentration and ionic strength for both COD removal and power density are the same. Maximum COD removal could be achieved with buffer concentration and ionic strength of 0.155 M and 4.34 mM, respectively.

### Optimization and verification

The optimal condition was predicted by “Numerical Optimization” toolbox of the Design Expert software version 7.0.0. The following constraints are exerted to obtain the optimal conditions, pH: 6.7-6.8, catholyte concentration: 0.17-0.18 M and ionic strength: 4.68-4.84 mM. The values of predicted optimal power density and COD removal are 32.5 mW/m^2^ and 92.5%, respectively at the optimal condition.

**Figure 6:**
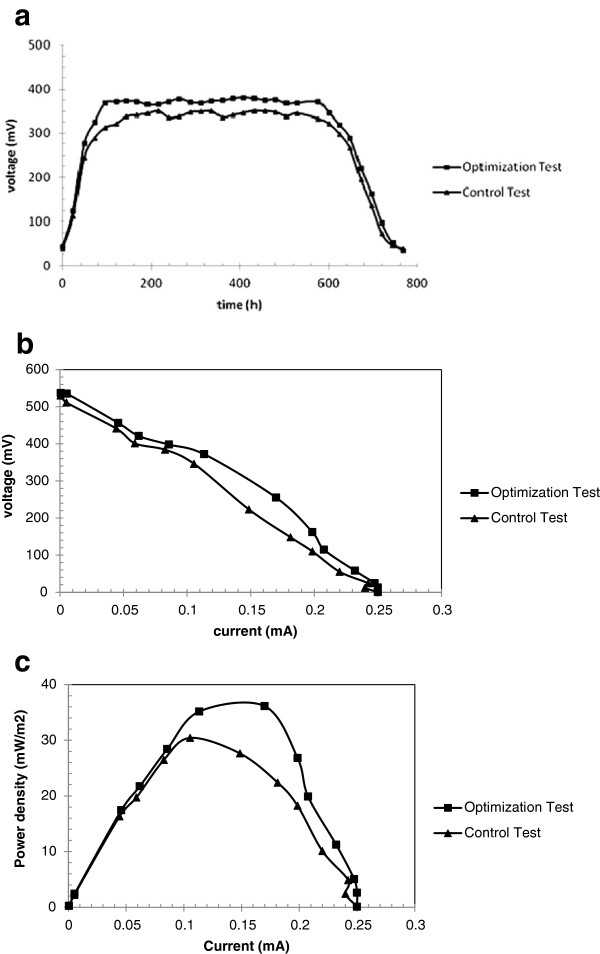
Optimization and control test, voltage versus time (a), polarization curve (b) and power-current curve (c).

To confirm the predicted optimum condition and its output by Design Expert, new experiments with the optimal conditions (test #1) and a control test under the zero level of independent variables (test #2) were conducted with the same MFC. For optimal conditions, two similar experiments were used to avoid any errors. The MFCs ran under the optimal condition (pH of 6.75, catholyte concentration of 0.17 M and ionic strength of 4.76 mM) and the control test ran under general condition (pH of 7, buffer concentration of 0.15 M and ionic strength of 5 mM) in batch mode. MFC’s voltage across a 3300 Ω resistor versus time, polarization and power-current curve were plotted for optimal and control condition in Figure 6a-c. COD of the anode compartments were determined from the start up to voltages below 50 mV. Later, average maximum output voltage of 372.5 and 345 mV were obtained in stationary phase of microorganism growth for test #1 and #2 respectively.

The power density in stationary phase was calculated and reached its maximum for the test #1. Average power density and COD removal were respectively 35 mW/m^2^ and 93.5% for optimum condition. On the other hand, power density and COD removal were 30 mW/m^2^ and 89.5% respectively for test #2. Thus, power density and COD removal were improved 17% and 5% respectively. Finally, the results verify that the optimum condition predicted by Design Expert could successfully improve MFC output. Moreover, the results confirm that the selected condition ranges are acceptable.

## Discussion

In this study effect of cathode operational parameters such as pH, catholyte concentration and ionic strength on MFC performance was evaluated and optimal operational condition was determined by response surface methodology. pH of cathode compartment selected as one of the most important factors which affect performance of microbial fuel cell. There are many researches that consider pH of anode chamber and its effects on power density and COD removal in microbial fuel cell [20,21] while just few study were performed on the impact of cathode chamber pH on MFC performance. In two chamber air cathode microbial fuel cell with catholyte pH equal to 1.0, power density was increased 2.5 fold compared to the same MFC with catholyte pH of 7.5 [22].

In addition, in air cathode MFC lower pH was preferred [23]. Power density of MFC with cathode compartment pH of 2.0 was increased 3.8 times higher than the power density obtained in the same MFC working at neutral pH. The better performance of MFC with low-pH cathode was likely due to that low pH guarantees high concentration of protons. Therefore, the concentration loss, as a cause of the cathodic overpotential, can be largely reduced under low pH condition. As a result of higher protons concentration in low pH, furthermore, the requirement of proton transfer through membrane was decreased and consequently, this would result in a decrease in ohmic loss.

Consequently, optimized pH of cathode chamber in present optimization study was equal to 6.75 in which power density and COD removal reached the maximum values. It is noteworthy that low pH of cathode chamber solution leads to reverse proton concentration gradient, reverse proton transfer, anode chamber acidification and microorganism death.

The higher concentration of cathode catholyte led to higher buffer capacity and kept the pH constant in the cathode compartment. Buffer concentration, moreover, affects membrane and catholyte resistance. Then, by using different phosphate buffer concentration (0.01, 0.03, 0.05 mM), membrane resistance were 230, 32.6 and 10.5 Ω respectively [24]. Membrane and catholyte resistance decreased significantly when higher concentration of buffer was used. Similarly, it was found that concentration of 0.175 M of phosphate buffer was improved MFC performance. Higher buffer concentration, although, improve power production and COD reduction in MFC as a result of higher conductivity and constant pH, while very high buffer concentration might lead to power density reduction because of precipitation of salt on electrode and membrane.

Ionic strength similar to buffer concentration has the same influence on conductivity. Consequently, the higher the ionic strength, the higher conductivity of catholyte and thus higher power produced in MFC. As a result, catholyte with 4.76 mM NaCl was determined that has the best effect on MFC performance. Moreover, effect of ionic strength on anode chamber has similar manner [8]. On the other hand, addition of NaCl to the anode compartment led to an increase in conductivity, and power production. Yet, higher concentration of NaCl (more than 10 mM) led to deactivation of microorganisms and finally, the concentration of 100 mM led to microorganism death.

## Conclusions

Application of response surface methodology (RSM) to determine the optimal condition of cathode compartment for maximizing power density and COD removal was successful. The optimal conditions were determined for pH, buffer concentration and ionic strength as 6.75, 0.175 M and 4.76 mM, respectively. In comparison with the normal condition (zero level), improvement of power density by 17% as well as 5% higher COD removal obtained by the system with optimal condition.

## Competing interests

The authors declare that they have no competing interests.

## Authors’ contributions

This work is part of the Ph.D. thesis of MH where MV and IA supervised the thesis. All authors read and approved the final manuscript.
